# PP97 Insulin Analogs In Type 1 Diabetes: Why Are There Barriers To Its Access In The Brazilian Unified Health System?

**DOI:** 10.1017/S0266462325103139

**Published:** 2025-12-29

**Authors:** Luiza Nascimento-Costa, Rosangela Caetano, Ione Ayala Gualandi Oliveira, Ione Oliveira

## Abstract

**Introduction:**

Despite being incorporated into the Brazilian Unified Health System (SUS) in 2017 and 2019, insulin analogs are still not well distributed to the populations who need them. These drugs are frequently the target of lawsuits in Brazil, which should be mitigated following the favorable recommendation of the National Committee for Health Technology Incorporation (CONITEC). This study aimed to understand the reasons for this delay in supplying the Brazilian population.

**Methods:**

This exploratory, descriptive, and retrospective research collected information from CONITEC’s website, purchases of insulin analogs by the Brazilian Ministry of Health registered in the General Services Administration’s Integrated System (integrated into Brazil’s Federal Purchasing Portal) and dispensing of medicines in the Outpatient Information System between 2011 and 2022. As a key variable, the study focused on the time between the incorporation decree and the first dispensation in the SUS, which should ideally occur within 180 days.

**Results:**

The time between the incorporation decree publication for short-acting insulin analogs and their first dispensation within the SUS was 575 days, 395 days longer than anticipated. In the case of long-acting insulin analogs, there were no scheduled purchases or drug dispensations after the incorporation until December 2024. The possible reasons for these delays include prolonged purchasing processes due to the need to meet specific requirements, shortages of insulin analogs in both national and global markets, conflicts among Brazilian federal entities responsible for financing and purchasing insulin analogs, and a lack of short-acting insulin analogs in the Ministry of Health’s stock, among others.

**Conclusions:**

It is essential to actively monitor the post-incorporation process of insulin analogs in Brazil due to the multiple barriers involved. This is particularly urgent because, as of November 2024, the public incorporation of this drug class, which was previously limited to treating patients with type 1 diabetes mellitus, has been extended to include patients with type 2 diabetes.

